# Resilience of the prokaryotic microbial community of *Acropora digitifera* to elevated temperature

**DOI:** 10.1002/mbo3.478

**Published:** 2017-04-20

**Authors:** Andrian P. Gajigan, Leomir A. Diaz, Cecilia Conaco

**Affiliations:** ^1^ Marine Science Institute University of the Philippines Diliman Quezon City Philippines

**Keywords:** coral, holobiont, microbiome, mucus, thermal stress

## Abstract

The coral is a holobiont formed by the close interaction between the coral animal and a diverse community of microorganisms, including dinoflagellates, bacteria, archaea, fungi, and viruses. The prokaryotic symbionts of corals are important for host fitness but are also highly sensitive to changes in the environment. In this study, we used 16S ribosomal RNA (rRNA) sequencing to examine the response of the microbial community associated with the coral, *Acropora digitifera,* to elevated temperature. The *A. digitifera* microbial community is dominated by operational taxonomic unit (OTUs) affiliated with classes *Alphaproteobacteria* and *Gammaproteobacteria*. The prokaryotic community in the coral tissue is distinct from that of the mucus and the surrounding seawater. Remarkably, the overall microbial community structure of *A. digitifera* remained stable for 10 days of continuous exptosure at 32°C compared to corals maintained at 27°C. However, the elevated temperature regime resulted in a decrease in the abundance of OTUs affiliated with certain groups of bacteria, such as order *Rhodobacterales*. On the other hand, some OTUs affiliated with the orders *Alteromonadales*,* Vibrionales,* and *Flavobacteriales,* which are often associated with diseased and stressed corals, increased in abundance. Thus, while the *A. digitifera* bacterial community structure appears resilient to higher temperature, prolonged exposure and intensified stress results in changes in the abundance of specific microbial community members that may affect the overall metabolic state and health of the coral holobiont.

## 1 Introduction

Coral reefs are among the most diverse and economically important ecosystems on the planet. A healthy coral is crucial to the productivity and sustainability of reef ecosystems and their surrounding communities (Riegl, Bruckner, Coles, Renaud, & Dodge, 2009). Corals serve as a habitat for a diverse community of symbionts, including dinoflagellates (*Symbiodinium*), fungi, bacteria, archaea and viruses. These symbionts have emerged as critical players in coral nutrition and health. Thus, maintaining symbiotic interactions is crucial for the functioning of the holobiont and its ability to adapt to environmental perturbation (Ainsworth & Gates, 2016; Bourne, Morrow, & Webster, 2016; Thompson, Rivera, Closek, & Medina, 2014). Understanding how each member of the holobiont contributes to the resilience of the organism is paramount in light of the rapidly changing ocean conditions brought about by global warming and anthropogenic impacts. These disturbances lead to coral bleaching events that consequently result in a decline in coral cover and loss of biodiversity (Hoegh‐Guldberg, 1999; Palumbi, Barshis, Traylor‐Knowles, & Bay, 2014).

A key to the survival of corals to the changing climate is their ability to adapt to environmental perturbation. Interestingly, certain corals are found in areas that are normally exposed to warmer or more variable temperatures. These corals have evolved mechanisms that provide greater tolerance to temperature stress (Guest et al., 2012; Thompson & van Woesik, 2009), such as the abundant expression of protective proteins (Barshis et al., 2013) and association with thermally tolerant *Symbiodinium* clades (Hume et al., 2015; Keshavmurthy et al., 2014). These corals may similarly be expected to maintain a thermally tolerant microbiome consisting of microorganisms that can support coral holobiont health even at elevated temperatures. However, it is important to note that although elevated temperature is a recognized stressor for corals, this might not be the case for mesophilic coral‐associated microbes that normally thrive under these temperatures.

Microbiome studies have revealed the diversity of bacteria associated with different coral host species (Dinsdale et al., 2008; Littman, Willis, Pfeffer, & Bourne, 2009; Morrow, Moss, Chadwick, & Liles, 2012; Rohwer, Breitbart, Jara, Azam, & Knowlton, 2001; Sunagawa, Woodley, & Medina, 2010). Here, we refer to the microbiome as the prokaryotic microorganisms (bacteria and archaea) associated with the coral host. Coral‐associated microorganisms are niche‐partitioned in the coral mucopolysaccharide layer, tissues, gastric cavity, and skeleton (Ainsworth et al., 2015; Rohwer, Seguritan, Azam, & Knowlton, 2002). The coral mucus layer, which forms a permeable barrier between the coral tissue and seawater, is the first line of defense against biofouling, pathogen invasion, and sedimentation (Brown & Bythell, 2005; Ritchie, 2006). Mucus is colonized by distinct microbial communities (Brown & Bythell, 2005) and may serve as a trap for nutrient‐bearing particles that can be ingested by the coral (Krediet, Ritchie, Paul, & Teplitski, 2013; Wild et al., 2004). The mucus layer, together with the coral immune response (van de Water et al., 2015), can prevent colonization of coral surfaces. However, the suppression of host defenses and induction of coral pathogen motility under conditions of stress may allow invasion of coral tissues (Garren, Son, Tout, Seymour, & Stocker, 2015).

While the full functional complexity of the coral‐associated microbiota is not yet well‐established, it has been shown that the stable association of the coral with *Symbiodinium* and bacteria provides access to nutrients and metabolic products and contributes to the energy budget of the host (Ainsworth, Fine, Blackall, & Hoegh‐Guldberg, 2006; Dobretsov & Qian, 2004; Lema, Willis, & Bourne, 2012; Lesser, Bythell, Gates, Johnstone, & Hoegh‐Guldberg, 2007). In the oligotrophic environment of the reef, the presence of multiple pathways for efficient assimilation of essential nutrients, such as carbon and nitrogen, provides an advantage. Microbes associated with corals are able to utilize organic carbon and fix inorganic nitrogen to support the metabolic requirements of the coral and *Symbiodinium* (Brown & Bythell, 2005; Cardini et al., 2015; Ceh, van Keulen, & Bourne, 2013; Lema et al., 2012). In addition, coral‐associated bacteria may play a role in controlling the growth of other microorganisms or preventing corals from being infected by pathogens (Bourne et al., 2016; Raina, Tapiolas, Willis, & Bourne, 2009; Rosenberg, Koren, Reshef, Efrony, & Zilber‐Rosenberg, 2007). Furthermore, bacteria have also been demonstrated to have the ability to regulate settlement and metamorphosis of corals (Hadfield, 2011; Negri, Webster, Hill, & Heyward, 2001; Webster et al., 2004).

The coral‐associated bacteria community is relatively resilient to fluctuations in the environment. However, perturbations will likely affect members of the community in different ways. For example, the microbial community in *Acropora millepora* was found to remain consistent during different times of the year but bleaching of the coral resulted in the appearance of *Vibrio*‐affiliated sequences (Bourne, Iida, Uthicke, & Smith‐Keune, 2008; Littman et al., 2009). *Acropora aspera* and *Stylophora pistillata* similarly exhibited variation in their microbial communities following coral bleaching (Ainsworth & Hoegh‐Guldberg, 2009). Interestingly, the bacterial community of *Acropora tenuis* associated with a thermotolerant clade of *Symbiodinium* was more sensitive to thermal perturbation compared to the bacterial community in *A. tenuis* hosting *Symbiodinium* from a different clade (Littman, Bourne, & Willis, 2010). This is most likely explained by the enhanced susceptibility of corals to opportunistic pathogens when *Symbiodinium*‐host interactions are sub‐optimal. This further suggests that, aside from the microbial community associated with coral tissue and mucus, the bacteria associated with *Symbiodinium*, particularly those with the ability to fix nitrogen (Lema et al., 2012), may also influence the resilience of corals to thermal stress. In *Acropora hemprichii* and *Mussismilia harttii*, the nitrogen‐fixing bacteria community increased in abundance or exhibited greater activity at elevated temperatures (Cardini et al., 2016; Santos et al., 2014). Thus, in order to better understand the influence of prokaryotic symbionts on coral physiology and health, it is important to elucidate the phylogenetic and functional diversity of the coral‐associated microbial community and its dynamic responses to the environment.

In this study, we demonstrate the effects of an elevated temperature regime on the association of prokaryotes with the common Indo‐Pacific scleractinian coral, *Acropora digitifera*. *A. digitifera* is a common Indo‐Pacific coral that is found in areas where temperatures can reach close to the upper limit of the thermal optimum for coral growth (Hoegh‐Guldberg, 1999; Veron, 2000). We reveal how shifts in the abundance of certain bacterial families may be linked to a decline in coral health and the enhancement of opportunistic bacteria associated with coral disease.

## 2 Materials and methods

### 2.1 Coral collection and maintenance

Three colonies of *Acropora digitifera* were collected from Bolinao, Pangasinan, Philippines (16° 17′ 28.6′’ N, 120° 00′ 44.2′’ E) at 2–4 m depth in February 2015. Collections were conducted with permission from the Philippines Department of Agriculture Bureau of Fisheries and Aquatic Resources (DA‐BFAR GP‐0102‐15). Coral colonies were fragmented into 2‐inch long nubbins (20 fragments per colony) and attached to reef plugs using epoxy. Reef plugs were labeled to enable tracking of the source colony of each fragment. The fragments were allowed to recover for a period of about 7 weeks in a tank with flowing seawater maintained at a temperature of 27 ± 1°C, average salinity of 33.6 PSU, pH of 8.11, and a 12‐hr light‐dark cycle with irradiance of 14 μmol m^−2^ s^−1^.

### 2.2 Thermal stress exposure

Thermal stress experiments were conducted in 40L tanks containing constantly aerated, flowing, sand‐filtered seawater and a 12 hr light‐dark cycle with irradiance of 14 μmol m^−2^ s^−1^. Seawater temperature was manipulated using submersible thermostat heaters. Tanks were monitored using temperature probes (Vernier Labquest 2) and submersible HOBO pendant temperature and light loggers (Onset). A low irradiance was used to reduce the potential contribution of high light intensity to the coral stress response (Downs et al., 2013). Following acclimatization, five fragments from each of the three colonies were transferred into two control and two treatment tanks (15 fragments per tank). Treatment tanks were maintained at 5°C above ambient (32 ± 1°C). Control tanks were maintained at 27 ± 1°C, representing the average temperature during the coldest months of the year (December to February) based on regular monitoring by the Bolinao Marine Laboratory. Throughout the course of the experiment, the state of the photosynthetic apparatus of the coral fragments was monitored, using a diving pulse‐amplitude‐modulated (PAM) fluorometer (Walz). PAM readings were taken from all coral fragments in experimental and control tanks. Coral and tissue fractions from fragments from each of two colonies from the same control or treatment tank were subjected to 16S rRNA sequencing after 10 days of exposure.

### 2.3 DNA extraction

Individual coral fragments were rinsed in membrane‐filtered seawater (FSW) then sealed in a 50 ml tube for 3 min to collect mucus secretions (Koren & Rosenberg, 2006; Meron et al., 2010). 400 μl of mucus was used for DNA extraction. After mucus collection, the coral fragments were rinsed several times in FSW to remove excess mucus. Coral tissues were then collected by dispersal into FSW using a WaterPik. Fifty ml of the tissue homogenate was spun at 664 x g for 15 min at 25°C to collect the tissue fraction. 1 L of seawater was also sampled from each experimental tank and the microbial fraction was collected by filtration through a 0.2 μm polycarbonate filter. DNA was extracted from mucus, tissue, and seawater samples using a modified CTAB method (Winnepenninckx, Backeljau, & De Wachter, 1993). Briefly, samples were mixed with CTAB extraction buffer (100 mmol/L TrisCl, pH 8.0, 20 mmol/L EDTA, 2% CTAB, 1.4M NaCl, 2.5 mg/ml lysozyme) and incubated at 37°C for 40 min. After addition of 0.2% β‐mercaptoethanol and 0.1 mg/ml proteinase K, samples were incubated at 60°C for 1 hr followed by chloroform fractionation and isopropanol precipitation. The DNA pellet was washed with 70% ethanol and dried at room temperature. The DNA was dissolved in 1x TE buffer and stored at −20°C.

### 2.4 16S ribosomal RNA (rRNA) sequencing and data analysis

Bacterial 16S rRNA was amplified, using barcoded primers (515F/806R) targeting the V4 hypervariable region (Caporaso et al., 2010). Paired‐end sequencing (250 bp) was performed on an Illumina MiSeq (BGI, Hongkong) following the dual‐index sequencing strategy (Kozich, Westcott, Baxter, Highlander, & Schloss, 2013). Briefly, 30 ng of genomic DNA was used for the PCR library preparation and sequencing. V4 dual‐index fusion PCR primer cocktail and PCR master mix (NEB Phusion High‐Fidelity PCR Master Mix) were used at a melting temperature of 56°C and 30 PCR cycles. The PCR products were then purified with AmpureXP beads (AGENCOURT) to remove unspecified products. The average molecule length was determined using Agilent 2100 Bioanalyzer and quantified using quantitative PCR. Sequence data is available on NCBI as project number PRJNA341929. Operational taxonomic unit (OTU) analysis of sequences was done, using the mothur v1.36.1 (Schloss et al., 2009). Size and quality filtering of reads were conducted as follows. Forward and reverse reads were merged to form a contig. Contigs with ambiguous bases and those that are >275 bp in length were removed. The assembled contigs were aligned to the SILVA database (release 102) (Quast et al., 2013). Chimeric sequences were checked and subsequently removed using the Uchime algorithm (Edgar, Haas, Clemente, Quince, & Knight, 2011). Sequences matching to chloroplast, eukaryotes, and mitochondria were also removed. Representative sequences were assigned taxonomic ranks using the Ribosomal Database Project (RDP) training set 16 with copy number adjustment (Wang, Garrity, Tiedje, & Cole, 2007). The extent of similarity between samples was assessed by weighted Unifrac analysis with a *p*‐value cutoff of 0.05 (Lozupone & Knight, 2005). The similarity of community structure between control and treated samples was assessed using ʃ‐Libshuff, analysis of molecular variance (AMOVA) and homogeneity of molecular variance (HOMOVA) (Schloss, 2008; Singleton, Furlong, Rathbun, & Whitman, 2001). Libraries were considered significantly different if the *p*‐value was <0.05 for AMOVA and HOMOVA or if either of the two *p*‐values generated for an individual pairwise comparison was <0.025 (ʃ‐Libshuff). Metastats was used to detect differentially abundant features (*p*‐value <0.05) in the sequence libraries (White, Nagarajan, & Pop, 2009). LEfSe, implemented in mothur, was used to detect bacterial groups that most likely explain differences between coral compartments (mucus and tissue) and seawater using an alpha value of 0.05 for both the Wilcoxon and Kruskal Wallis ANOVA tests and an LDA score threshold of 2.0 (Segata et al., 2011). Calculation of Chao1 and inverse Simpson diversity indices, as well as the LEfSe and Metastats analyses, were conducted using subsampled libraries to keep the number of sequences the same across samples. Subsampling was done by random selection of sequences based on the size of the smallest library.

## 3 Results

### 3.1 Diversity of the coral‐associated microbial community

To understand how the coral‐associated microbial community responds to sustained exposure to elevated temperature, the V4 hypervariable region of the 16S rRNA gene was sequenced using Illumina Miseq. Duplicate samples of tissue and mucus from corals exposed to 27°C and 32°C, as well as seawater samples from experimental tanks, were included in the analysis. At an average sequencing depth of 69,107 reads per sample, the rarefaction curve for coral tissue reached saturation, whereas the curves for coral mucus and seawater communities did not, indicating that the latter samples were composed of more complex communities (Figure [Link], [Link]). A total of 13,286 operational taxonomic units (OTUs) were identified at a 97% sequence identity cutoff. About 2,468 and 2,498 OTUs were detected in the control and treated mucus samples, respectively, while only 1,220 and 1,166 OTUs were found in control and treated tissue samples, respectively (Figure [Link], [Link]). Coral OTUs were grouped into approximately 33 phyla, 57 classes, and 83 different bacterial and archaeal orders. It is important to note, however, that in silico evaluation of 525F/806R V4 primers using SILVA TestPrime (Klindworth et al., 2013) reveals that only 52.6% archaea and 86.2% bacteria will be amplified. In addition to the primer coverage limitation, this primer set results in underrepresentation of the SAR11 clade (Apprill, McNally, Parsons, & Weber, 2015) and *Thaumarchaeota* (Parada, Needham, & Fuhrman, 2016) and limits detection of phyla under candidate phyla radiation (CPR) (Brown et al., 2015). Hence, we speculate that utilization of other primer sets will detect an even greater number of OTUs.

**Figure 1:**
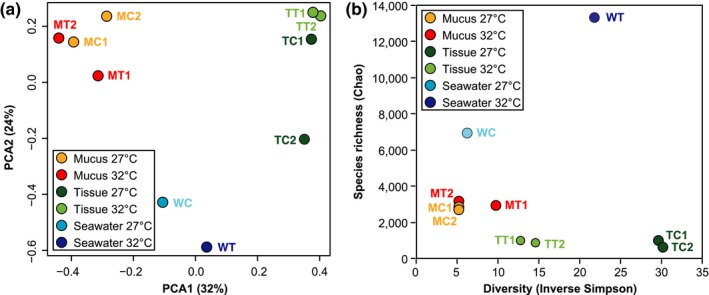
Comparison of bacterial communities in coral tissue, mucus, and seawater. (a) Principal component analysis based on the Yue & Clayton dissimilarity measure reveals that tissue, mucus, and seawater samples can be distinguished by their microbial community composition based on 16S rRNA phylotypes (mucus 27°C, MC; mucus 32°C, MT; tissue 27°C, TC; tissue 32°C, TT; seawater 27°C, WC; seawater 32°C, WT). Numbers indicate the colony source for mucus and tissues samples. The percent of total variation explained by each component is shown in parentheses. (b) Seawater communities have the highest species richness, followed by coral mucus and tissue. Richness is based on the Chao1 index. On the other hand, seawater and coral tissues have higher species diversity compared to mucus communities. Diversity is based on the Inverse Simpson index

The bacterial community in the tissues and mucus layer of *A. digitifera* is distinct from the bacteria found in surrounding seawater (Figure 1a). Water and mucus samples exhibited higher species richness compared to coral tissues. In contrast, tissue and water samples exhibited higher diversity compared to the mucus (Figure 1b).

**Figure 2:**
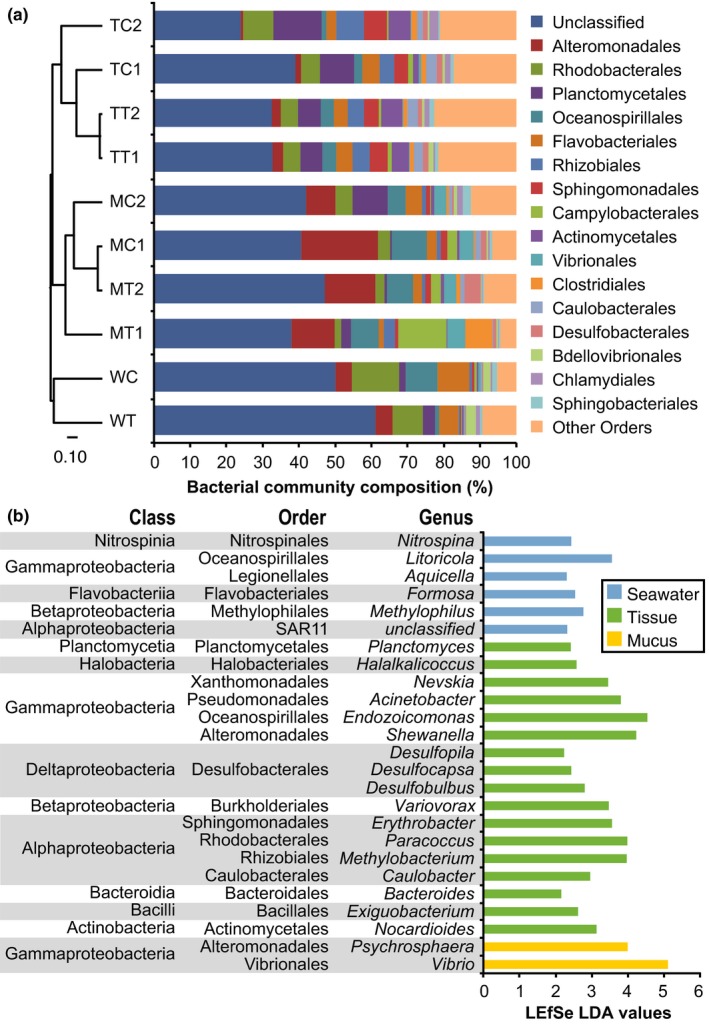
Bacterial community composition of coral tissue, mucus, and seawater. (a) Relative abundance of bacterial taxa classified to order level and corrected for copy number. The dendrogram is based on the Yue & Clayton measure of dissimilarity. (b) Bacterial taxa that distinguish between coral tissue, mucus, and seawater communities based on LEfSe analysis (LDA>2, alpha < 0.05)

Similar to previous observations in other corals (Bayer et al., 2013; Blackall, Wilson, & van Oppen, 2015; Bourne et al., 2016; Li et al., 2014; Littman et al., 2009), the majority of the identified OTUs in *A. digitifera* belong to the *Proteobacteria* phylum and is dominated by the *Gammaproteobacteria* and *Alphaproteobacteria* classes (Figure [Link], [Link]). The *Gammaproteobacteria* make up 13% of the community in coral tissue, 45% in mucus, and 21% in seawater. *Gammaproteobacteria* orders that are found at higher proportion in coral mucus include *Alteromonadales*,* Oceanospirillales*, and *Vibrionales* (Figure 2a). Class *Alphaproteobacteri*a makes up 24% of the tissue community, 12% of mucus, and 18% of seawater. *Alphaproteobacteria* orders that are found at higher proportion in coral tissue include *Rhizobiales*,* Rhodobacterales*, and *Spingomonadales*. Order *Actinomycetales* and *Flavobacteriales* are also found at higher proportion in coral tissue. The *Endozoicomonas* genus of *Gammaproteobacteria*, which can constitute up to 90% of the bacterial community of certain corals (Bayer et al., 2013; Morrow et al., 2012; Rodriguez‐Lanetty, Granados‐Cifuentes, Barberan, Bellantuono, & Bastidas, 2013), only makes up 1.2–15.1% of the *Gammaproteobacteria* in *A. digitifera*. LEfSe analysis further revealed bacterial groups that distinguish between seawater, coral tissue, and coral mucus (Figure 2b). Seawater has a greater abundance of OTUs affiliated with *Nitrospinales, Oceanospirillales* (genus *Litoricola*), *Flavobacteriales*, and SAR11. OTUs belonging to *Planctomycetales, Pseudomonadales, Oceanospirillales* (genus *Endozoicomonas*), *Alteromonadales* (genus *Shewanella*), *Desulfobacterales, Rhodobacterales, Rhizobiales, Bacteroidales, Bacillales*, and *Actinomycetales*, are enriched in coral tissue. OTUs belonging to order *Alteromonadales* (genus *Psychrosphaera*) and *Vibrionales* (genus *Vibrio*) are enriched in coral mucus.

### 3.2 Response of the coral‐associated microbial community to elevated temperature

Sustained exposure of *A. digitifera* fragments to elevated temperature did not result in obvious bleaching. However, on the tenth day of exposure, we observed a slight but significant decline in photosynthetic efficiency (Fv/Fm) from 0.82 ± 0.04 to 0.70 ± 0.05 (Student's *t*‐test, *p* < 0.001) in the coral fragments at 32°C relative to the controls maintained at 27°C (Figure [Link], [Link]). This indicates some level of deterioration of the cellular integrity of photosynthetic dinoflagellate symbionts within the coral tissues. It is likely that continued exposure to elevated temperature would eventually result in breakdown of the coral‐algal symbiosis (Brown, 1997; Kvitt, Rosenfeld, Zandbank, & Tchernov, 2011).

Prolonged exposure to 32°C did not appear to cause any large‐scale changes in coral bacterial community structure compared to controls at 27°C based on OTU composition analysis using ʃ‐Libshuff, HOMOVA, and AMOVA (Table S1 and S2). Coral communities remained dominated by *Proteobacterial* classes. While the total number of OTUs detected in coral samples exposed to elevated temperatures was similar to samples that were maintained at 27°C, shifts in the relative frequency of specific OTUs were detected (weighted UniFrac, *p* < 0.05). Sustained exposure to elevated temperature resulted in significant changes in the abundance of 209 unique OTUs in coral tissue and mucus (Metastats, *p* < 0.05) (Table S3). The majority of differentially abundant OTUs in coral tissues showed an increase in relative abundance (74%, 125 of 168 OTUs). In contrast, a larger proportion of differentially abundant OTUs in coral mucus decreased (71%, 32 of 45 OTUs) (Table S3).

### 3.3 Bacterial groups affected by elevated temperature exposure

**Figure 3:**
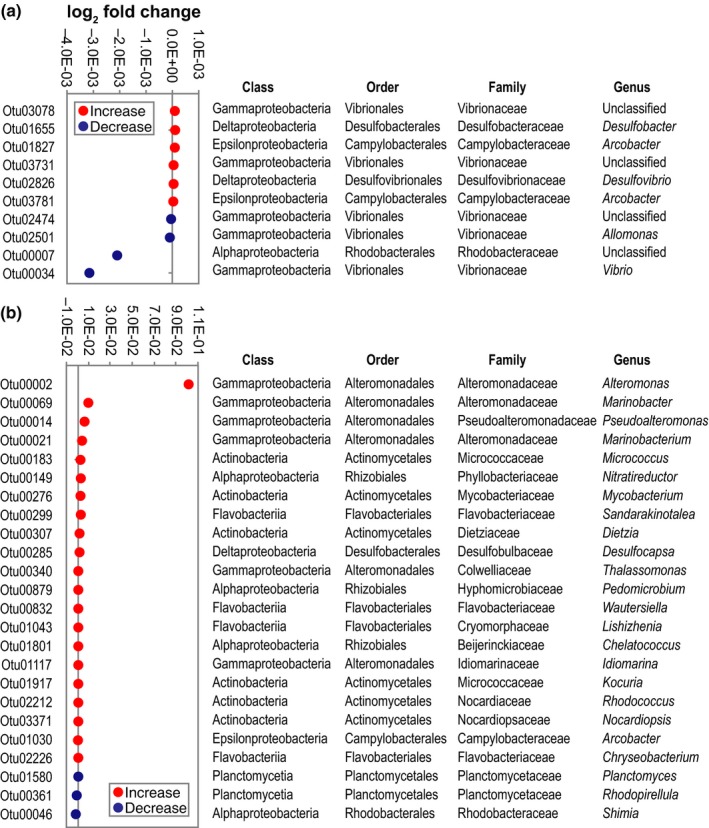
Thermal stress results in changes in the abundance of specific bacterial groups. Plot representing the relative abundance (log_2_ fold change) of OTUs in coral mucus (a) or tissue (b) that are significantly different between samples exposed to 32°C or 27°C based on Metastats analysis (*p* < 0.05). Red circles represent OTUs that increased in frequency while blue circles represent OTUs that decreased in frequency

Exposure to elevated temperature resulted in a change in the frequency of several bacterial groups associated with the tissue and mucus of *A. digitifera* (Figure 3; Table S3). Most OTUs belonging to order *Rhodobacterales* (class *Alphaproteobacteria*) exhibited a significant decrease in abundance in both coral mucus and tissue. On the other hand, most OTUs belonging to order *Rhizobiales* (class *Alphaproteobacteria)* increased in coral tissue. In coral mucus, some OTUs belonging to order *Vibrionales* (class *Gammaproteobacteria)* increased while some decreased in abundance. OTUs belonging to order *Alteromonadales* (class *Gammaproteobacteria)* increased in coral tissue. *Deltaproteobacteria* OTUs, which include the sulfate‐reducing *Desulfobacterales* and *Desulfovibrionales,* also increased in abundance in coral mucus and tissue. Other notable groups that exhibited a significant increase in abundance in coral tissues exposed to elevated temperatures include OTUs affiliated with *Actinobacteria* and *Epsilonproteobacteria*, specifically the genus *Arcobacter*. Most *Planctomycetia* OTUs decreased in abundance in coral tissue.

## 4 Discussion

### 4.1 Coral‐associated microbiota

The coral microbial community described here includes members of the bacterial groups reported to represent the coral core microbiome (Ainsworth et al., 2015; Bourne et al., 2016), as well as groups that have been previously detected in other corals. Among the commonly reported coral bacteria are members of order *Rhodobacterales (*class *Alphaproteobacteria),* order *Actinomycetales* (class *Actinobacteria*)*,* and genus *Endozoicomonas* (class *Gammaproteobacteria*). Some bacterial groups found in the *A. digitifera* microbiome, including *Oceanospirillales, Alteromonadales*, and *Vibrionales*, have been implicated in the metabolism of dimethylsulfoniopropionate (DMSP) that is produced by photosymbionts, as well as in the fixation of nitrogen (Bourne et al., 2013; Raina et al., 2009). Moreover, the order *Oceanospirillales,* which is dominant in the coral mucus, has been reported to be vertically transmitted within coral larvae and has a symbiotic role in coral development (Bayer et al., 2013; Speck & Donachie, 2012). The characteristic functional properties of these different bacterial groups may shape their symbiotic relationship with the host coral.

### 4.2 Distinct microbial communities in coral compartments exhibit varying responses

There are distinct differences in the association of bacterial groups with different coral compartments, which may reflect variable conditions within each micro‐niche. For instance, excretion of broadly active compounds in the mucus allows the host to effectively control the microbiota and to detect pathogenic microorganisms or trigger defense responses by exclusion of undesirable community members or selection of symbionts (Krediet, Ritchie, Paul et al., 2013). Chemical cues or nutrients in the mucus may also attract microorganisms with potentially beneficial functions, or attract and maintain keystone microbes that would in turn shape the microbiota to be resistant to invasion by potential pathogens. Alternatively, it is possible that some members of the bacterial community can alter their physiology to take advantage of the warmer environment. In fact, the bacteria may themselves initiate their release from the coral surface through a change in their lifestyle or motility properties, perhaps enhancing their ability to invade other niches (Garren & Azam, 2011; Garren et al., 2015; Ritchie & Smith, 2004).

We found that mucus samples exhibit lower diversity than tissue. Because diversity indices take into account both species richness and evenness, the lower diversity index of mucus samples may reflect greater number of rare OTUs. The presence of rare bacterial groups in the mucus communities is expected given the potential for microbial exchange at this interface between the coral and the surrounding seawater (Lema et al., 2012). Exposure to thermal stress resulted in a decrease in microbial diversity in coral tissue although species richness remained almost the same, signaling a change in the abundance of various bacteria within this compartment. Also, it is observed that more OTUs significantly increased in proportion in the tissue, which may be due to migration or proliferation of bacteria in coral tissues. More OTUs in the mucus decreased in proportion, which is likely due to the shedding of the mucus layer, changes in mucus composition, or depletion of mucus reserves, which can directly affect carbon cycling within the mucus microniche (Garren & Azam, 2011; Lee, Davy, Tang, & Kench, 2016). The release of mucus is part of the mechanism by which the coral‐associated bacterial population may be controlled, as has been previously described in the coral *Acropora eurystoma* exposed to varying pH levels (Meron et al., 2010) and corals exposed to organic enrichment (Garren & Azam, 2011).

### 4.3 Response of coral‐associated microbes to thermal stress

Interestingly, few bacterial groups associated with *A. digitifera* exhibited a significant response to elevated temperature exposure. Nevertheless, some of these taxa are potentially important in terms of their effects on holobiont metabolism or host health. For example, *Alteromonadales* and *Vibrionales*, which changed in abundance in coral tissue and mucus under elevated temperature conditions, have previously been linked to coral stress and disease (Bourne, Iida, Uthicke, & Smith‐Keune, 2007; Garren & Azam, 2011; Garren, Raymundo, Guest, Harvell, & Azam, 2009; Krediet, Ritchie, Paul, et al., 2013). Although not all *Vibrio* are pathogenic, some species have been reported to be responsible for certain coral diseases associated with increased sea surface water temperatures (Ben‐Haim et al., 2003; Kimes et al., 2012). Members of the genera *Alteromonas* and *Vibrio* are common marine bacteria that possess a diverse metabolic repertoire allowing them to exploit the nutrient rich micro‐niches of corals (Lopez‐Perez et al., 2012; Thompson, Iida, & Swings, 2004). Both *Alteromonadales* and *Vibrionales* have been recovered from the coral surface mucus layer where the microbiota interacts with potential pathogens and environmental organisms (Littman et al., 2010). However, the bacteria may also be able to migrate between the mucus and tissue compartments (Garren et al., 2015; Lee, Davy, Tang, Fan, & Kench, 2015). Interestingly, isolates of these bacteria taken from various corals exhibit antimicrobial properties (Shnit‐Orland & Kushmaro, 2009) that may confer a competitive advantage against other members of the microbial community.

In contrast to observations from thermal stress experiments in the coral *Acropora muricata* (Lee et al., 2015), where total *Gammaproteobacteria* decreased while *Verrucomicrobiaceae* and total *Alphaproteobacteria* increased, we observed a general decrease in the relative abundance of *Alphaproteobacteria* OTUs and an increase in total *Gammaproteobacteria* OTUs in *A. digitifera*. Members of *Alphaproteobacteria*, specifically order *Rhodobacterales,* are ubiquitous in coral reef ecosystems (Lawler et al., 2016) and comprise one of the dominant orders of bacteria in corals (Kemp et al., 2015) while order *Rhizobiales* are potentially *Symbiodinium*‐associated taxa (Ainsworth et al., 2015). It has been shown that *Alphaproteobacteria* are typically associated with reefs that have higher coral cover while *Gammaproteobacteria* and *Flavobacteriales* are abundant in more degraded algae‐dominated reefs (Kelly et al., 2014). The dominance of *Gammaproteobacteria* in the mucus microbiome of corals at the Bolinao reef complex may partly reflect the long‐term influence of multiple stressors, including highly variable temperature (Peñaflor, Skirving, Strong, Heron, & David, 2009) and high nutrient input from fish farming activities in the area (Garren et al., 2009). Differences in host specificity and geographic variability may also contribute to the observed differences in the response of the microbial community of *A. digitifera* and *A. muricata* to elevated temperature (Hester, Barott, Nulton, Vermeij, & Rohwer, 2016; McKew et al., 2012; Morrow et al., 2012; Neave et al., 2017).

The *Deltaproteobacteria*, which include the sulfate‐reducing bacteria *Desulfobacterales* and *Desulfovibrionales*, increased in abundance in treated relative to the control samples. Sulfate‐reducing bacteria are present in healthy corals but have also been implicated as part of the microbial consortium that induces Black Band Disease (Arboleda & Reichardt, 2008; Meron et al., 2010). We speculate that depletion of oxygen as the temperature rises triggers an increase in the abundance of sulfate reducers. Furthermore, the genus *Arcobacter* of *Epsilonproteobacteria* increased in corals under stress. Members of this group are closely related to human and animal pathogens (Miller et al., 2007) but have also been isolated from diverse marine environments (Kim, Hwang, & Cho, 2009; Wirsen et al., 2002). Most OTUs under the *Bacteroidetes* group, specifically order *Flavobacteriales,* increased in tissue fractions of corals exposed to higher temperature. *Flavobacteriales* have been detected in the corals *Orbicella faveolata* (Sunagawa et al., 2009) and *Oculina patagonica* (Koren & Rosenberg, 2006) and are associated with White Band Disease in *Acropora cervicornis* (Gignoux‐Wolfsohn & Vollmer, 2015), Black Band Disease, and other stress conditions of scleractinian corals (Frias‐Lopez, Zerkle, Bonheyo, & Fouke, 2002; Meron et al., 2010; Sekar, Kaczmarsky, & Richardson, 2008; Thurber et al., 2009).

*Actinobacteria* were also found to increase in *A. digitifera* tissues under stress. Members of this group are known to produce compounds with antibacterial activities. Thus, their increased abundance during stress may be key to controlling the coral bacterial population and preventing the invasion of potential pathogens (Krediet, Ritchie, Alagely, & Teplitski, 2013; Ritchie, 2006). Diverse *Actinobacteria* communities have been reported in many corals, including *A. digitifera* from the Gulf of Mannar (Nithyanand, Indhumathi, Ravi, & Pandian, 2011), *Porites lutea* from the South China Sea (Kuang, Li, Zhang, & Long, 2015), and other corals from the thermally stressed reefs of the Arabian Gulf (Mahmoud & Kalendar, 2016). In addition, the decrease in abundance of most *Planctomycetia* OTUs, which are associated to nitrogen fixation or degradation of sulfated polymers (Glockner et al., 2003; Zehr, Mellon, & Zani, 1998), may contribute to the reduction in these metabolic capacities within the holobiont.

The shift in microbial community under elevated temperature conditions suggests that the coral‐associated bacteria have differing sensitivities to thermal stress. The diversity of the microbial community in corals provides functional redundancy as various members have the potential to access alternative metabolic pathways for survival that may be critical for adaptability of the holobiont during conditions of stress. Although some groups represented within the coral have been previously associated with disease states, it remains unclear whether the bacterial types that significantly change in abundance under conditions of elevated temperature represent pathogens driving diseased states or are merely opportunists taking advantage of the shift in bacterial assemblage or of host physiology.

## 5 Conclusions

The importance of the microbial symbionts for the survival of *A. digitifera* is underscored by the finding that the genome of this coral lacks a key enzyme for the synthesis of the amino acid cysteine, which is crucial for proper folding of protein structures and is often involved in enzymatic reactions (Shinzato et al., 2011). This coral is thus likely dependent on its symbionts for the provision of cysteine and the precursors for its synthesis. Our findings, however, show that *A. digitifera* and its associated microbiota can respond dynamically to the environment as an essential mechanism of coping with stress. Although specific members of the coral bacterial community may have greater sensitivity, the maintenance of the general community structure even after prolonged exposure to elevated temperature underscores the resilience of this community to higher temperatures. The key to the stability of the bacterial community of *A*. *digitifera* may lie in its diversity and functional redundancy, with multiple species contributing to the metabolic demands of the host through their ability to access alternative metabolic pathways that may be critical for adaptability of the holobiont during conditions of stress.

However, despite the ability of the bacterial community to adapt to local conditions through the selection of advantageous metabolic genes (Kelly et al., 2014), sustained environmental stress will impact nutrient cycling and have lasting effects on the holobiont defense systems with the loss of community‐mediated growth control mechanisms.** **This emphasizes the importance of maintaining the structure of the community, as disturbances that disrupt the natural abundance of certain bacteria may allow opportunistic members to proliferate or pathogenic bacteria to invade coral tissues (Krediet, Ritchie, Alagely, et al., 2013; Lee et al., 2016). The ability of the coral‐associated bacterial community to rapidly shift in response to external conditions can either exacerbate the effects of stress or support the rapid adaptation of corals to the changing environment (Reshef, Koren, Loya, Zilber‐Rosenberg, & Rosenberg, 2006).

Changes in the microbial community could directly reflect shifts in environmental parameters and could be used to detect changes in coral fitness in response to the environment. Understanding the connections between certain bacterial groups with nutrient cycling potential or pathogenic effects will contribute further insights into the role of the microbiome in the resilience of corals to stress and disease.

## Conflict of Interest

The authors declare no conflict of interest.
