# Supplementary material for: Resilience of the prokaryotic microbial community of Acropora digitifera to elevated temperature
Source: Microbiologyopen. 2017 Apr 20;6(4):e00478. doi: 10.1002/mbo3.478 (PMC5552946; doi:10.1002/mbo3.478)
Supplement: Supplementary file 1 [file MBO3-6-na-s001.pdf]

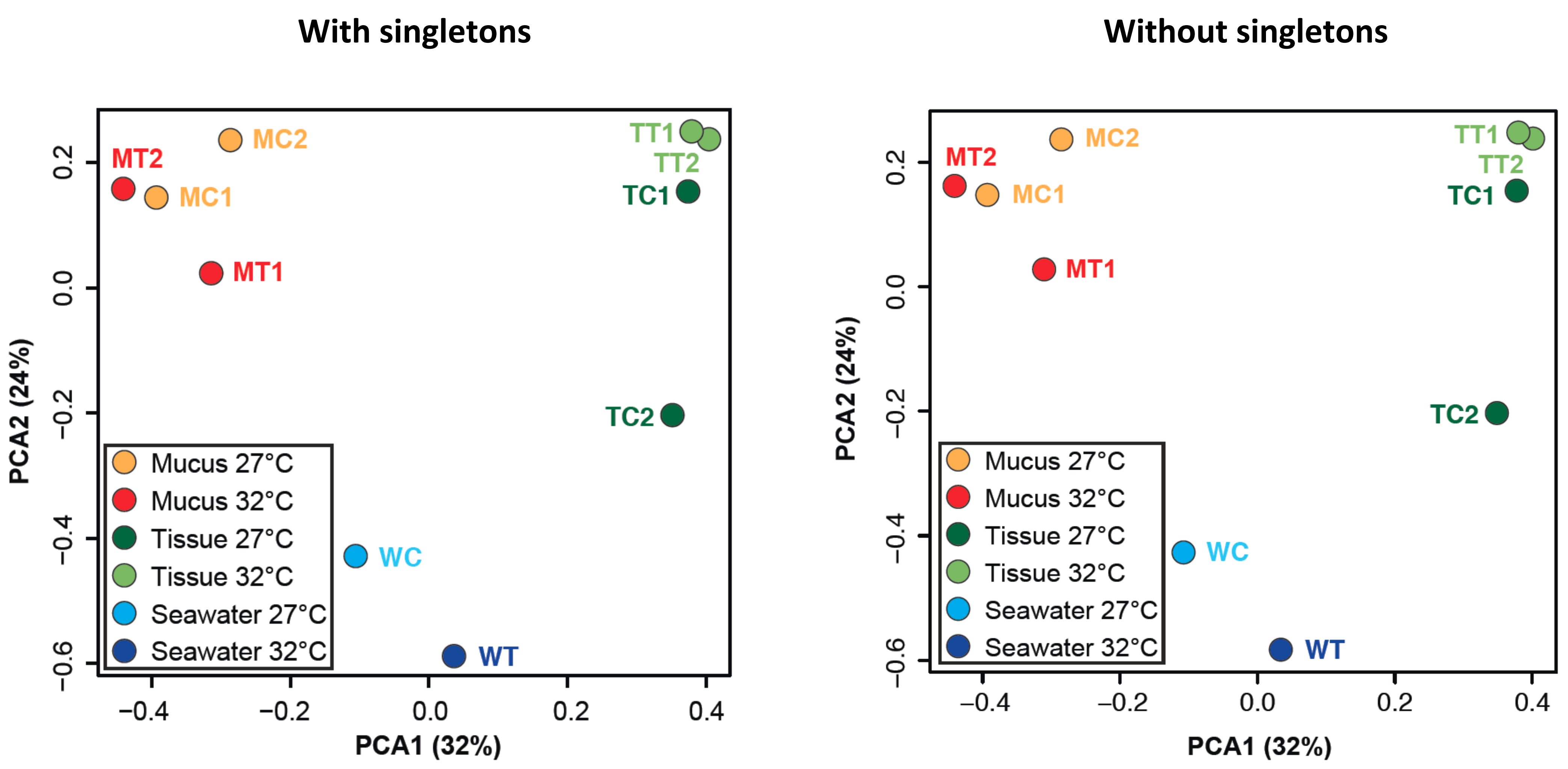

Figure 1. Principal component analysis based on the Yue & Clayton dissimilarity measure. The numbers represent coral colonies from which the sample was obtained. The percent of total variation explained by each component is shown in parentheses.

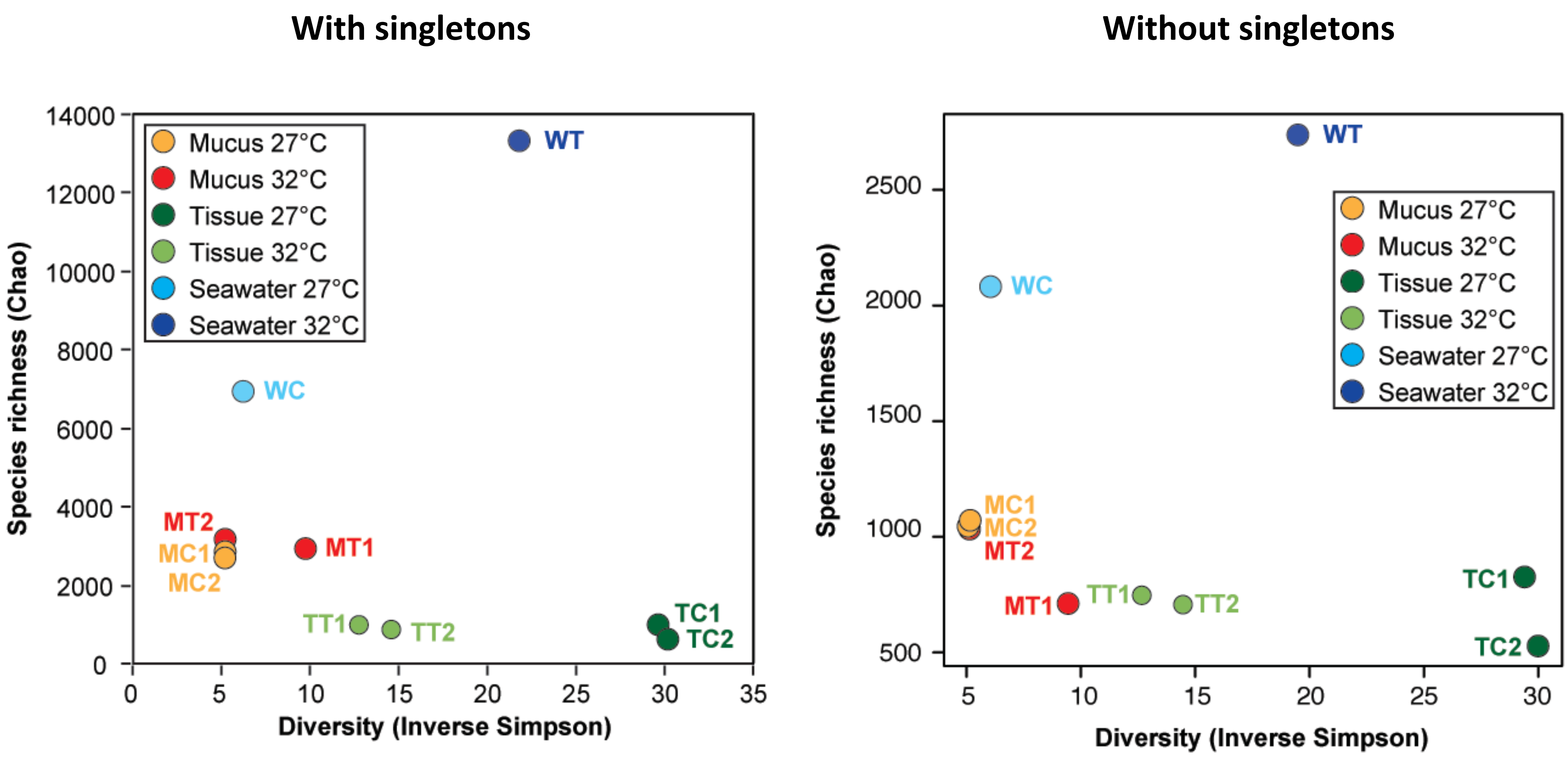

Figure 2. Comparison of richness and diversity among samples. Seawater communities have the highest species richness, followed by coral mucus and tissue. Richness is based on the Chao1 index. On the other hand, seawater and coral tissues have higher species diversity compared to mucus communities. Diversity is based on the Inverse Simpson index.

Mucus comparison

With singletons

Unweighted UniFrac							
thetayc				jclass			
Tree#	Groups	UWScore	UWSig	Tree#	Groups	UWScore	UWSig
1	MC vs MT	0.69	1.00	1	MC vs MT	0.97	1.00
**Weighted UniFrac**							
thetayc				jclass			
Tree#	Groups	WScore	WSig	Tree#	Groups	WScore	WSig
1	MC vs MT	0.55	0.05	1	MC vs MT	0.94	0.04
**AMOVA**							
thetayc				jclass			
MC vs MT	Among	Within	Total	MC vs MT	Among	Within	Total
SS	0.09	0.44	0.53	SS	0.37	0.87	1.24
df	1.00	2.00	3.00	df	1.00	2.00	3.00
MS	0.09	0.22		MS	0.37	0.44	
Fs:	0.39			Fs:	0.86		
p-value:	1.00			p-value	0.67		
**HOMOVA**							
thetayc				jclass			
	Bvalue	P-value			BValue	P-value	
MC vs MT	0.03	1.00		MC vs MT	0.00	0.68	
**∫-LIBSHUFF**							
thetayc				jclass			
Comparison	dCXYScore	Significance		Comparison	dCXYScore	Significance	
MC vs MT	0.24	0.50		MC vs MT	0.03	0.83	
MT vs MC	0.27	0.50		MT vs MC	0.07	0.34	

Without singletons

Unweighted UniFrac							
thetayc				jclass			
Tree#	Groups	UWScore	UWSig	Tree#	Groups	UWScore	UWSig
1	MC vs MT	0.69	1.00	1	MC vs MT	0.92	1.00
**Weighted UniFrac**							
thetayc				jclass			
Tree#	Groups	WScore	WSig	Tree#	Groups	WScore	WSig
1	MC vs MT	0.55	0.04	1	MC vs MT	0.86	<0.001
**AMOVA**							
thetayc				jclass			
MC vs MT	Among	Within	Total	MC vs MT	Among	Within	Total
SS	0.09	0.44	0.53	SS	0.30	0.80	1.09
df	1.00	2.00	3.00	df	1.00	2.00	3.00
MS	0.09	0.22		MS	0.30	0.40	
Fs:	0.39			Fs:	0.75		
p-value:	1.00			p-value	1.00		
**HOMOVA**							
thetayc				jclass			
	Bvalue	P-value			Bvalue	P-value	
MC vs MT	0.03	1.00		MC vs MT	0.002	0.64	
**∫-LIBSHUFF**							
thetayc				jclass			
Comparison	dCXYScore	Significance		Comparison	dCXYScore	Significance	
MC vs MT	0.24	0.67		MC vs MT	0.06	0.83	
MT vs MC	0.27	0.50		MT vs MC	0.11	0.34	

Figure 3. Statistical tests to determine whether the A. digitifera mucus microbial community at 32°C (MT) has similar structure to controls maintained at 27°C (MC) using Yue & Clayton (thetayc) and Jaccard (jclass) dissimilarity index as input.

Tissue comparison

With singletons

Unweighted UniFrac							
thetayc				jclass			
Tree#	Groups	UWScore	UWSig	Tree#	Groups	UWScore	UWSig
1	TC vs TT	0.86	0.35	1	TC vs TT	1.00	0.35
**Weighted UniFrac**							
thetayc				jclass			
Tree#	Groups	WScore	WSig	Tree#	Groups	WScore	WSig
1	TC vs TT	0.81	<0.001	1	TC vs TT	1.00	<0.001
**AMOVA**							
thetayc				jclass			
TC vs TT	Among	Within	Total	TC vs TT	Among	Within	Total
SS	0.35	0.23	0.58	SS	0.40	0.52	0.93
df	1.00	2.00	3.00	df	1.00	2.00	3.00
MS	0.35	0.12		MS	0.40	0.26	
Fs:	2.99			Fs:	1.53		
p-value:	0.322			p-value	0.322		
**HOMOVA**							
thetayc				jclass			
	Bvalue	P-value			BValue	P-value	
TC vs TT	3.70	0.33		TC vs TT	0.03	0.33	
**∫-LIBSHUFF**							
thetayc				jclass			
Comparison	dCXYScore	Significance		Comparison	dCXYScore	Significance	
TC vs TT	0.09	1.00		TC vs TT	0.02	1.00	
TT vs TC	0.45	0.17		TT vs TC	0.16	0.16	

Without singletons

Unweighted UniFrac							
thetayc				jclass			
Tree#	Groups	UWScore	UWSig	Tree#	Groups	UWScore	UWSig
1	TC vs TT	0.86	0.33	1	TC vs TT	1.00	0.32
**Weighted UniFrac**							
thetayc				jclass			
Tree#	Groups	WScore	WSig	Tree#	Groups	WScore	WSig
1	TC vs TT	0.81	<0.001	1	TC vs TT	1.00	<0.001
**AMOVA**							
thetayc				jclass			
TC vs TT	Among	Within	Total	TC vs TT	Among	Within	Total
SS	0.35	0.23	0.58	SS	0.39	0.47	0.86
df	1.00	2.00	3.00	df	1.00	2.00	3.00
MS	0.35	0.12		MS	0.40	0.24	
Fs:	2.99			Fs:	1.66		
p-value:	0.33			p-value	0.34		
**HOMOVA**							
thetayc				jclass			
	Bvalue	P-value			Bvalue	P-value	
TC vs TT	3.70	0.31		TC vs TT	0.04	0.35	
**∫-LIBSHUFF**							
thetayc				jclass			
Comparison	dCXYScore	Significance		Comparison	dCXYScore	Significance	
TC vs TT	0.09	1.00		TC vs TT	0.02	1.00	
TT vs TC	0.45	<0.0001		TT vs TC	0.19	0.17	

Figure 4. Statistical tests to determine whether the A. digitifera tissue microbial community at 32°C (TT) has similar structure to controls maintained at 27°C (TC) using Yue & Clayton (thetayc) and Jaccard (jclass) dissimilarity index as input.

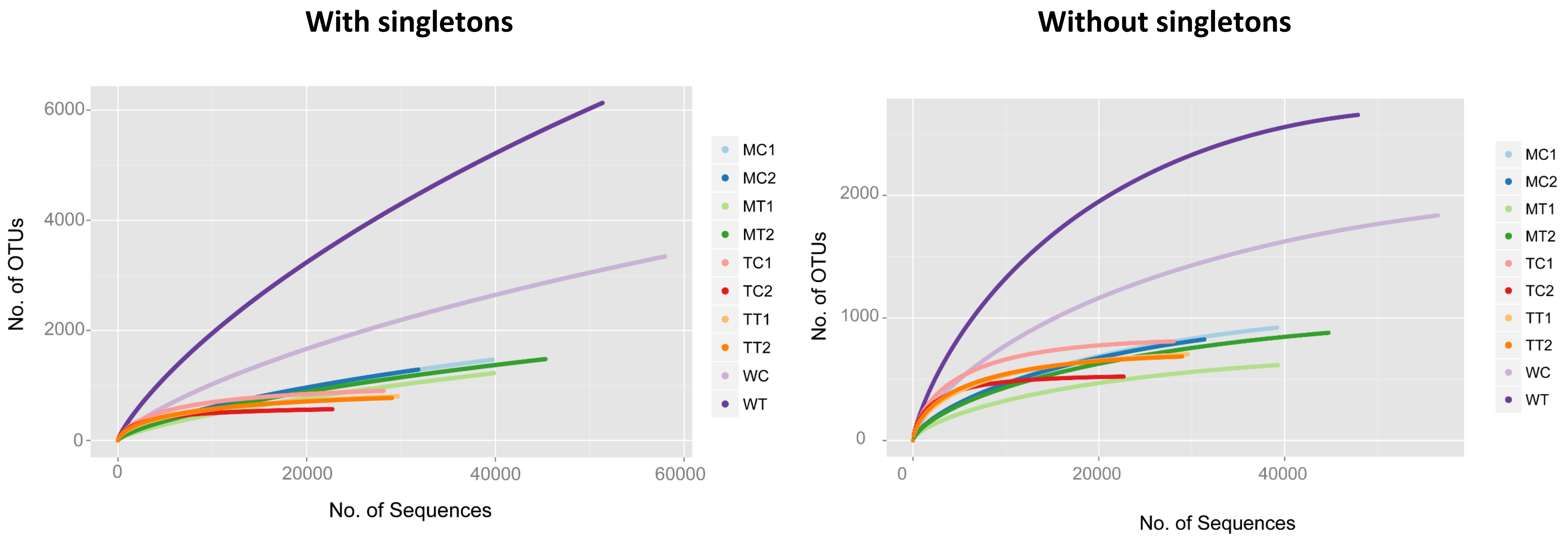

Figure 5. Rarefaction curves for all samples at 97% sequence similarity cut-off.

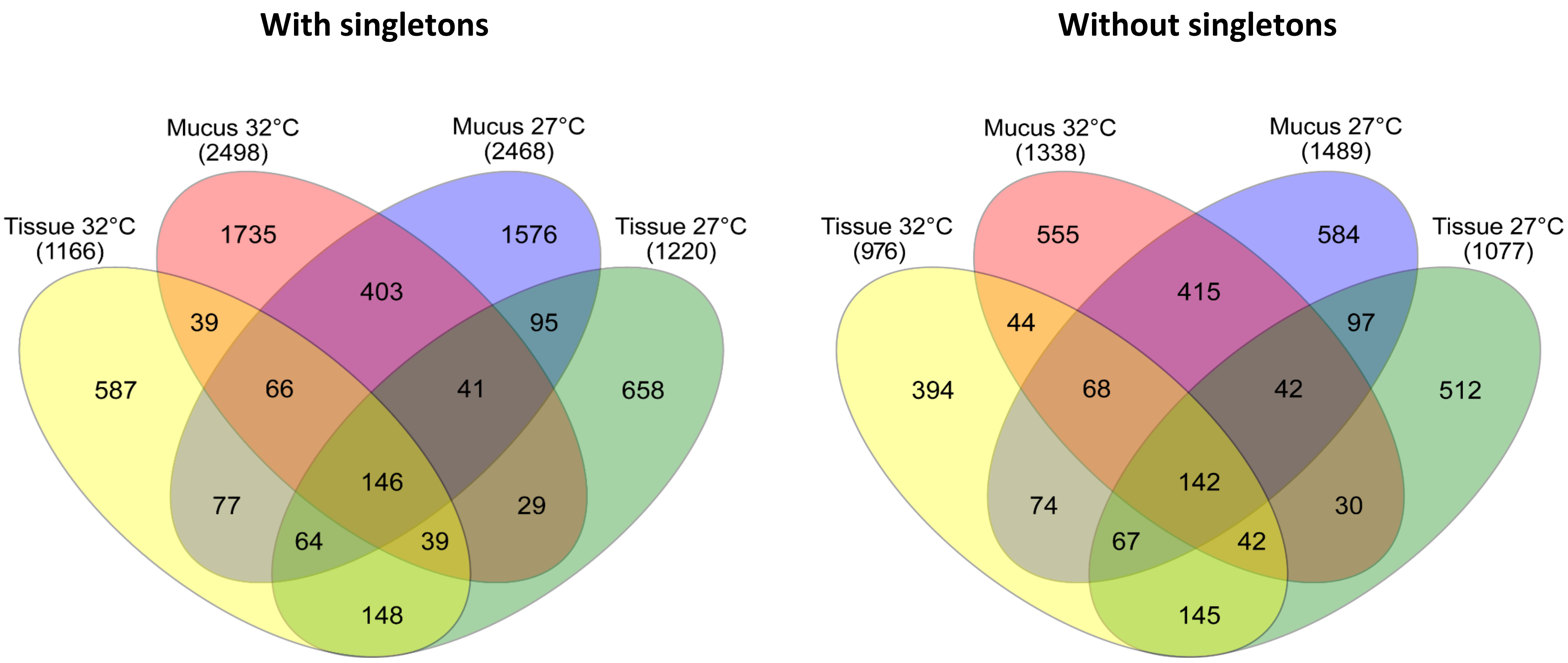

Figure 6. The number of common and unique OTUs identified in coral mucus and tissue after 10 days of exposure at 27°C and 32°C. The total number of OTUs identified in the sequence library for each fraction is shown in parenthesis.

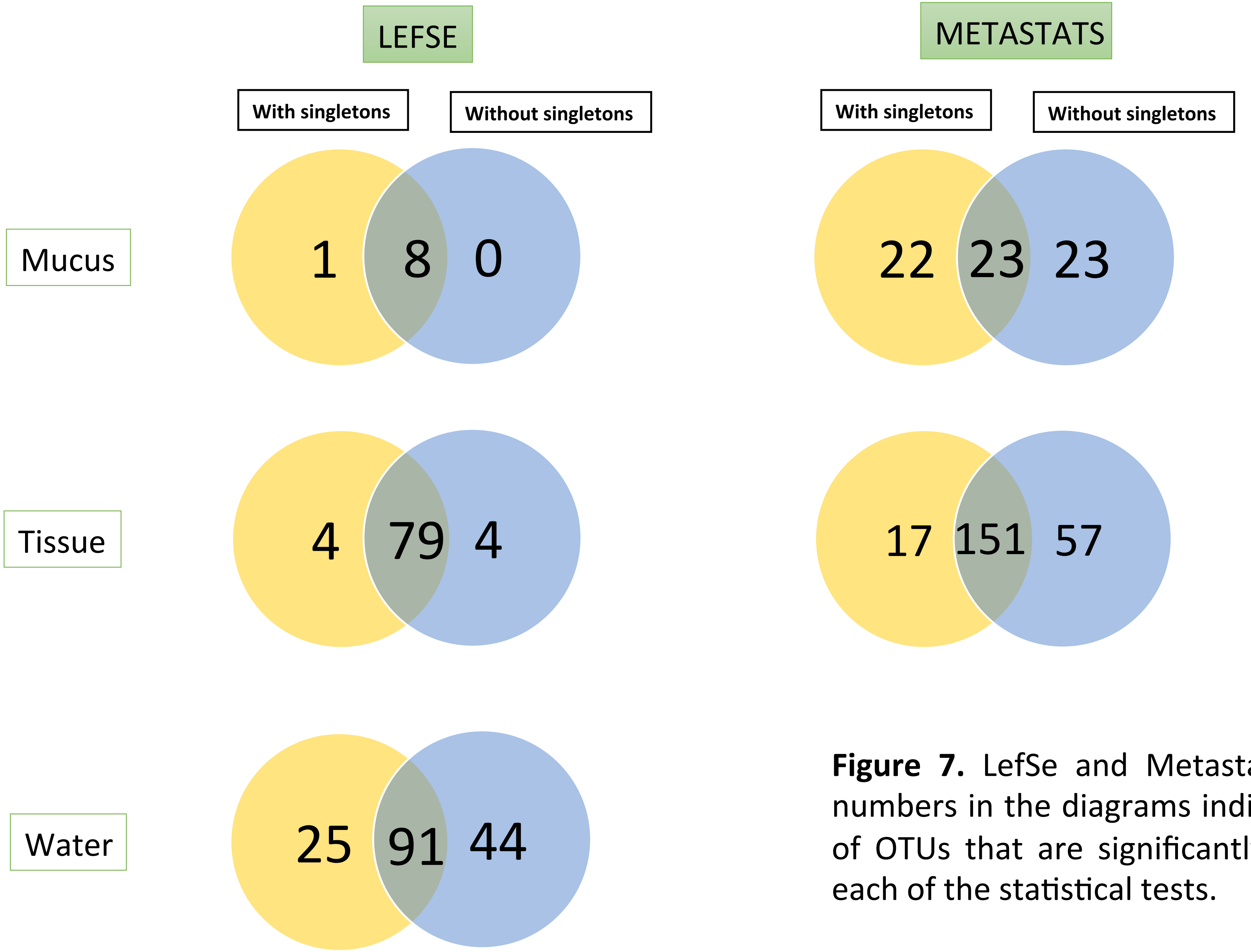

Figure 7. LefSe and Metastats analysis. The numbers in the diagrams indicate the number of OTUs that are significantly represented in each of the statistical tests.
