# Supplementary material for: Resilience of the prokaryotic microbial community of Acropora digitifera to elevated temperature
Source: Microbiologyopen. 2017 Apr 20;6(4):e00478. doi: 10.1002/mbo3.478 (PMC5552946; doi:10.1002/mbo3.478)
Supplement: Supplementary file 2 [file MBO3-6-na-s002.docx]

**Supplementary Information**


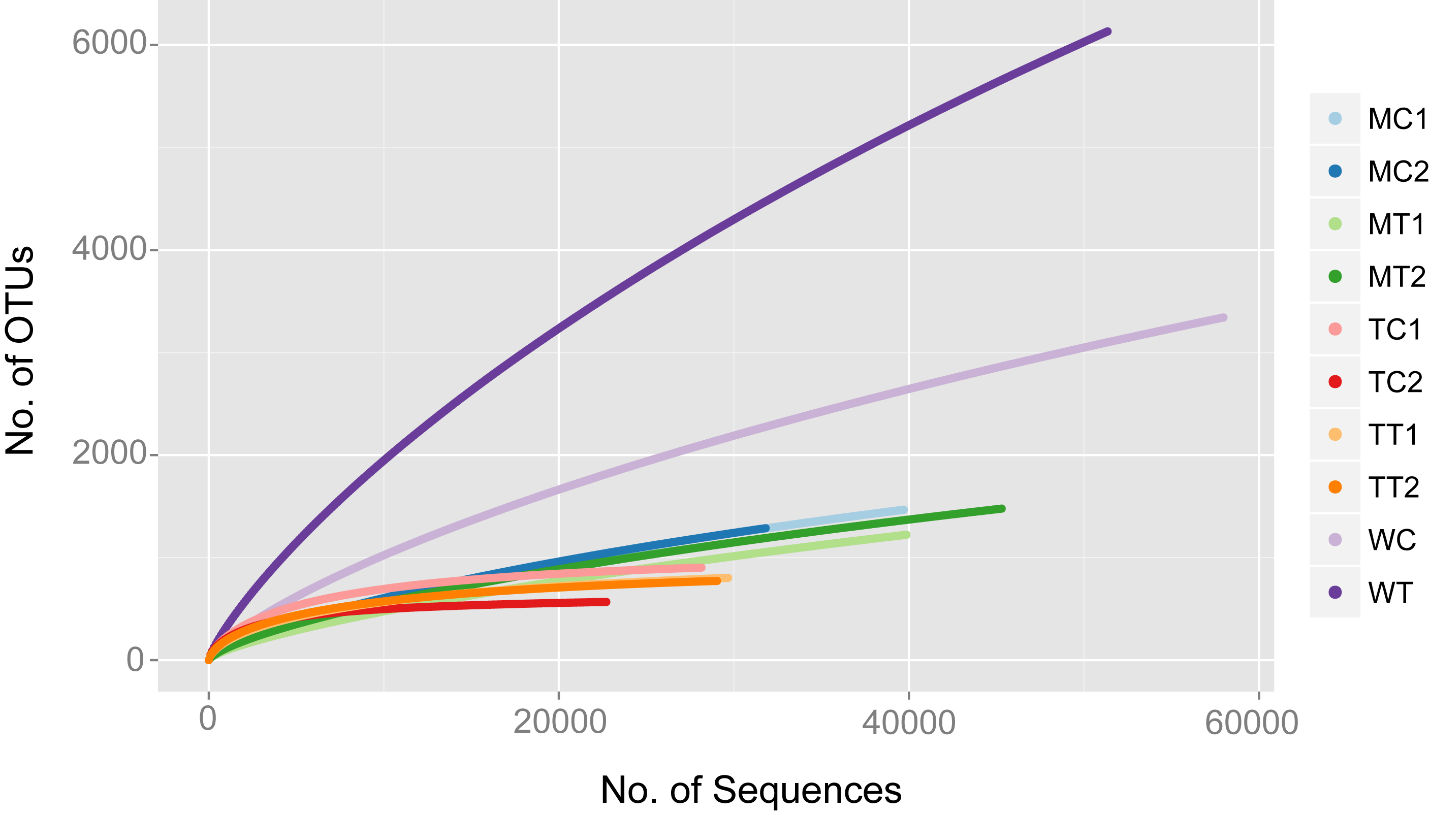


**Figure S1.** Rarefaction curves for all samples at 97% sequence similarity cut-off.


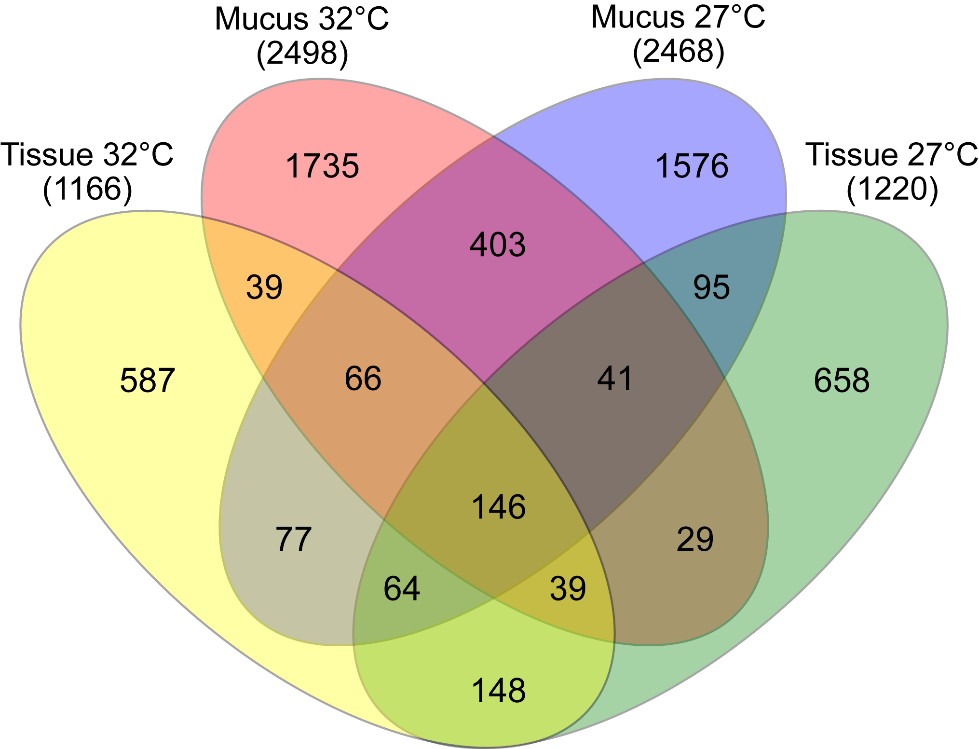


**Figure S2.** The number of common and unique OTUs identified in coral mucus and tissue after 10 days of exposure at 27°C and 32°C. The total number of OTUs identified in the sequence library for each fraction is shown in parenthesis.

**
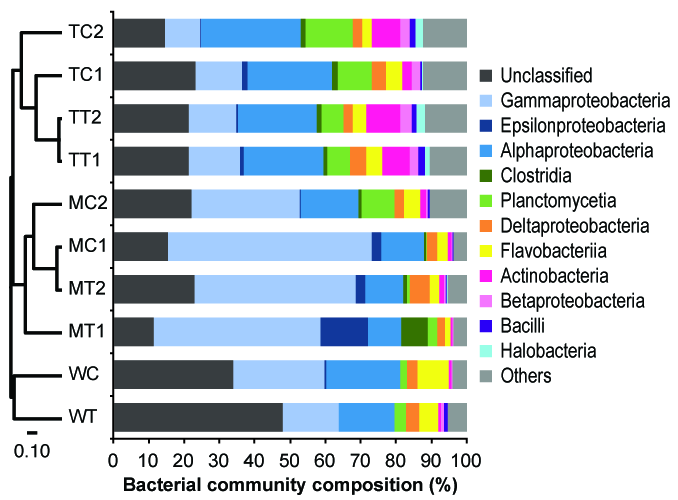
**

**Figure S3. Bacterial community composition of coral tissue, mucus, and seawater.** Relative abundance of bacterial taxa classified to class level.


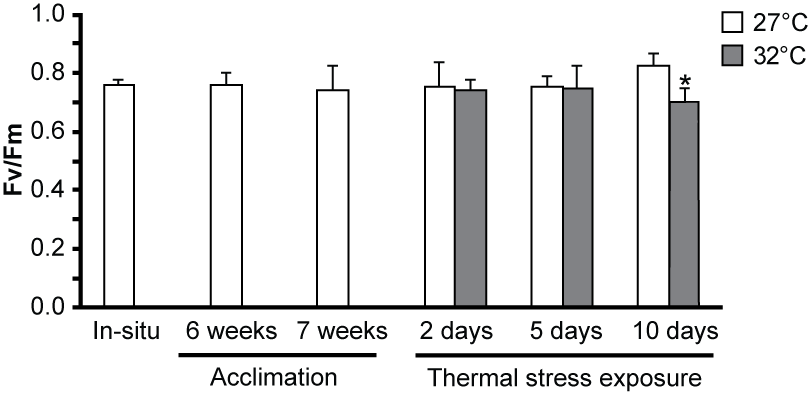


**Figure S4.** Photosynthetic efficiency of *Acropora digitifera* fragments. Photosystem II photochemical efficiency (Fv/Fm) was monitored during acclimation and during the thermal stress exposure at 32°C compared to controls at 27°C. The asterisk indicates a significant difference using two-tailed, paired, Student t-test (p<0.05).

**Table S1.** Statistical tests to determine whether the *A. digitifera* mucus microbial community at 32°C (MT) has similar structure to controls maintained at 27°C (MC) using Yue & Clayton (thetayc) and Jaccard (jclass) dissimilarity index as input.

| **Unweighted UniFrac** | | | | | | | |
| --- | --- | --- | --- | --- | --- | --- | --- |
| thetayc | | | | jclass | | | |
| Tree# | Groups | UWScore | UWSig | Tree# | Groups | UWScore | UWSig |
| 1 | MC vs MT | 0.69 | 1.00 | 1 | MC vs MT | 0.97 | 1.00 |
| **Weighted UniFrac** | | | | | | | |
| thetayc | | | | jclass | | | |
| Tree# | Groups | WScore | WSig | Tree# | Groups | WScore | WSig |
| 1 | MC vs MT | 0.55 | 0.05 | 1 | MC vs MT | 0.94 | 0.04 |
| **AMOVA** | | | | | | | |
| thetayc | | | | jclass | | | |
| MC vs MT | Among | Within | Total | MC vs MT | Among | Within | Total |
| SS | 0.09 | 0.44 | 0.53 | SS | 0.37 | 0.87 | 1.24 |
| df | 1.00 | 2.00 | 3.00 | df | 1.00 | 2.00 | 3.00 |
| MS | 0.09 | 0.22 |  | MS | 0.37 | 0.44 |  |
| Fs: | 0.39 |  |  | Fs: | 0.86 |  |  |
| p-value: | 1.00 |  |  | p-value | 0.67 |  |  |
| **HOMOVA** | | | | | | | |
| thetayc | | | | jclass | | | |
|  | Bvalue | P-value | |  | BValue | P-value | |
| MC vs MT | 0.03 | 1.00 | | MC vs MT | 0.00 | 0.68 | |
| **ʃ-LIBSHUFF** | | | | | | | |
| thetayc | | | | jclass | | | |
| Comparison | dCXYScore | Significance | | Comparison | dCXYScore | Significance | |
| MC vs MT | 0.24 | 0.50 | | MC vs MT | 0.03 | 0.83 | |
| MT vs MC | 0.27 | 0.50 | | MT vs MC | 0.07 | 0.34 | |

**Table S2.** Statistical tests to determine whether the *A. digitifera* tissue microbial community at 32°C (TT) has similar structure to controls maintained at 27°C (TC) using Yue & Clayton (thetayc) and Jaccard (jclass) dissimilarity index as input.

| **Unweighted UniFrac** | | | | | | | |
| --- | --- | --- | --- | --- | --- | --- | --- |
| thetayc | | | | jclass | | | |
| Tree# | Groups | UWScore | UWSig | Tree# | Groups | UWScore | UWSig |
| 1 | TC vs TT | 0.86 | 0.35 | 1 | TC vs TT | 1.00 | 0.35 |
| **Weighted UniFrac** | | | | | | | |
| thetayc | | | | jclass | | | |
| Tree# | Groups | WScore | WSig | Tree# | Groups | WScore | WSig |
| 1 | TC vs TT | 0.81 | <0.001 | 1 | TC vs TT | 1.00 | <0.001 |
| **AMOVA** | | | | | | | |
| thetayc | | | | jclass | | | |
| TC vs TT | Among | Within | Total | TC vs TT | Among | Within | Total |
| SS | 0.35 | 0.23 | 0.58 | SS | 0.40 | 0.52 | 0.93 |
| df | 1.00 | 2.00 | 3.00 | df | 1.00 | 2.00 | 3.00 |
| MS | 0.35 | 0.12 |  | MS | 0.40 | 0.26 |  |
| Fs: | 2.99 |  |  | Fs: | 1.53 |  |  |
| p-value: | 0.322 |  |  | p-value | 0.322 |  |  |
| **HOMOVA** | | | | | | | |
| thetayc | | | | jclass | | | |
|  | Bvalue | P-value | |  | BValue | P-value | |
| TC vs TT | 3.70 | 0.33 | | TC vs TT | 0.03 | 0.33 | |
| **ʃ-LIBSHUFF** | | | | | | | |
| thetayc | | | | jclass | | | |
| Comparison | dCXYScore | Significance | | Comparison | dCXYScore | Significance | |
| TC vs TT | 0.09 | 1.00 | | TC vs TT | 0.02 | 1.00 | |
| TT vs TC | 0.45 | 0.17 | | TT vs TC | 0.16 | 0.16 | |
